# The prevalence and predictors of household food insecurity among adolescents in Canada

**DOI:** 10.17269/s41997-022-00737-2

**Published:** 2023-01-23

**Authors:** Ruojun Liu, Marcelo L. Urquia, Valerie Tarasuk

**Affiliations:** 1 Department of Nutritional Sciences, Temerty Faculty of Medicine, University of Toronto, Toronto, ON Canada; 2 Manitoba Centre for Health Policy, University of Manitoba, Winnipeg, MB Canada; 3 Institute for Clinical Evaluative Sciences, Toronto, ON Canada; 4 Dalla Lana School of Public Health, University of Toronto, Toronto, ON Canada

**Keywords:** Food insecurity, Adolescents, Logistic models, Canadian Community Health Survey, Insécurité alimentaire, adolescents, modèles logistiques, Enquête sur la santé dans les collectivités canadiennes

## Abstract

**Objectives:**

Household food insecurity is almost four times more prevalent among adolescents than among older adults in Canada, and it adversely affects their health. Our objective was to describe the sociodemographic and geographic patterning of household food insecurity among adolescents.

**Methods:**

Our analytic sample comprised all 12–17-year-old respondents to the 2017–2018 Canadian Community Health Survey with complete data on household food insecurity (*n* = 8416). We used bivariate and multivariable logistic regression models to identify respondent- and household-level sociodemographic characteristics associated with household food insecurity.

**Results:**

The prevalence of household food insecurity among adolescents was 20.7%. The adjusted odds of food insecurity were significantly elevated among adolescents who identified as Black or Indigenous (aOR 1.80), those living with a single parent (aOR 1.60), those living with a greater number of children ≤ 5 years (aOR 1.45) or 12–17 years (aOR 1.25), those in rented accommodation (aOR 1.98), those in households with only secondary school education (aOR 1.38), and those in households reliant on social assistance (aOR 2.03). Higher before-tax income was protective (aOR 0.99). In comparison with Ontario, the adjusted odds of food insecurity among adolescents were higher in Nunavut (aOR 6.77), Northwest Territories (aOR 2.11), and Alberta (aOR 1.48), and lower in Manitoba (aOR 0.66).

**Conclusion:**

The markedly higher odds of exposure to household food insecurity among adolescents who are Black or Indigenous and those living in households characterized by markers of social and economic disadvantage highlight the need for more effective policy interventions to protect vulnerable families from this hardship.

**Supplementary Information:**

The online version contains supplementary material available at 10.17269/s41997-022-00737-2.

## Résumé

**Objectifs:**

L’insécurité alimentaire des ménages est près de quatre fois plus prévalente chez les adolescents que chez les personnes âgées au Canada, et elle nuit à leur santé. Nous avons cherché à décrire la structuration sociodémographique et géographique de l’insécurité alimentaire des ménages chez les adolescents.

**Méthode:**

Notre échantillon d’analyse comprenait tous les répondants de 12 à 17 ans du cycle 2017-2018 de l’Enquête sur la santé dans les collectivités canadiennes, ainsi que les données complètes sur l’insécurité alimentaire des ménages (n = 8 416). Nous avons utilisé des modèles de régression logistique bivariés et multivariés pour déterminer les caractéristiques sociodémographiques des répondants et des ménages associées à l’insécurité alimentaire des ménages.

**Résultats:**

La prévalence de l’insécurité alimentaire des ménages chez les adolescents était de 20,7 %. Le risque relatif rapproché d’insécurité alimentaire était significativement élevé chez les adolescents s’identifiant comme étant noirs ou autochtones (rapport de cotes ajusté [RCa] 1,80), vivant avec un seul parent (RCa 1,60), vivant avec un grand nombre d’enfants de ≤ 5 ans (RCa 1,45) ou de 12 à 17 ans (RCa 1,25), vivant dans des logements locatifs (RCa 1,98), vivant dans des ménages n’ayant fait que des études secondaires (RCa 1,38) et vivant dans des ménages dépendant de l’aide sociale (RCa 2,03). Le revenu élevé avant impôt était un facteur de protection (RCa 0,99). Comparativement à l’Ontario, le risque relatif rapproché d’insécurité alimentaire chez les adolescents était plus élevé au Nunavut (RCa 6,77), aux Territoires du Nord-Ouest (RCa 2,11) et en Alberta (RCa 1,48), et plus faible au Manitoba (RCa 0,66).

**Conclusion:**

Le risque nettement plus élevé d’exposition à l’insécurité alimentaire des ménages chez les adolescents noirs ou autochtones et chez ceux vivant dans des ménages caractérisés par les indicateurs de défavorisation sociale et économique montre que des interventions stratégiques plus efficaces sont nécessaires pour protéger les familles vulnérables de cette épreuve.

## Introduction

Household food insecurity, defined as inadequate or insecure access to food due to financial constraints, is a serious public health problem in many high-income countries, including Canada. This problem disproportionately affects families with children (Reeves et al., 2021). Based on the 2018 Canadian Community Health Survey, the most recent nationally representative data available, one in five 12–17 year-olds lived in a household affected by some level of food insecurity, compared to one in ten adults 50–64 years of age and almost one in twenty adults 65 and older (Polsky & Garriguet, 2022). Although the health implications of food insecurity for adolescents have not been studied as extensively as those for adults in this country, adolescents in food-insecure households in Canada have poorer quality dietary intakes (Hutchinson & Tarasuk, 2021), higher odds of mental health problems (Men et al., 2021a; Ovenell et al., 2022), and higher incidence of injuries (Men et al., 2021b) compared to adolescents in food-secure households. In the United States, where there has been considerably more research on the relationship between household food insecurity and the health of children and youth, food insecurity among adolescents has been shown to increase their odds of elevated blood pressure and prediabetes (Lee et al., 2019), dyslipidemia (Tester et al., 2016), iron-deficiency anemia (Eicher-Miller et al., 2009), and lower bone mass (Eicher-Miller et al., 2011).

The demographics of household food insecurity in Canada are well established. Independent predictors of food insecurity in the general population include low income; being reliant on social assistance, Employment Insurance (EI) or Workers’ Compensation; renting rather than owning one’s home; being a lone-parent female-led household or unattached individual; being Indigenous or Black; and not having a university education (Dhunna & Tarasuk, 2021; McIntyre et al., 2016b; Tarasuk et al., 2019a). In addition, lower rates of food insecurity have been repeatedly observed among households reliant on seniors’ income sources, a finding attributed to the protection afforded by the income adequacy and security provided to Canadian seniors through our public pension system (McIntyre et al., 2016a). There are also large jurisdictional differences in food insecurity rates, with the household prevalence ranging from 11.1% in Quebec to 57.0% in Nunavut in 2017–2018 (Tarasuk & Mitchell, 2020).

Given the high proportion of adolescents in Canada living in food-insecure households and the serious negative health implications of this exposure, it is important to understand which adolescents are most at risk. Such knowledge is key to setting priorities for intervention and developing effective responses. Drawing on data from the most recent, nationally representative population survey to measure food insecurity in Canada, our objective was to describe the sociodemographic and geographic patterning of household food insecurity among adolescents.

## Methods

### Data and study sample

We used data from the 2017–2018 cycle of the Canadian Community Health Survey (CCHS). Conducted by Statistics Canada, CCHS samples individuals 12 years of age and older living in Canada, excluding people living in institutions, full-time members of the Canadian forces, and those living on First Nation reserves or in one of two remote northern Quebec regions. Household food insecurity over the past 12 months is measured using the Household Food Security Survey Module (HFSSM), a validated 18-item scale of severity. We included all respondents aged 12 to 17 years (*n* = 8686) except those whose household food insecurity status could not be classified and the very small number who appeared to be living in a household without at least one individual aged 18 and older (*n* = 270). The final sample was 8416 adolescents.

### Measures

The outcome of interest was household food insecurity status over the past 12 months, defined using the classification scheme developed by Health Canada for the HFSSM (Government of Canada, 2020). Households with no affirmative response were considered “food secure”, and those with 1 or more affirmative responses were considered “food insecure”. Food-insecure households were further differentiated as marginally, moderately, or severely food insecure, with the latter two groups combined to preserve power.

We considered household- and respondent-level sociodemographic characteristics associated with household food insecurity in prior Canadian studies (Dhunna & Tarasuk, 2021; McIntyre et al., 2014; McIntyre et al., 2016b; Tarasuk et al., 2019a). These included respondent’s self-reported sex, ethno-racial identity (white, Black, Indigenous, other/missing), immigration status (Canadian born, immigrant < 5 years, immigrant ≥ 5 years), and living arrangement (living with a single parent, living with two parents, other/missing). Household characteristics included province/territory of residence, rural/urban residence, highest level of education in the household (less than secondary school graduation, secondary school graduation, post-secondary certification, missing), before-tax household income, main source of household income, housing tenure (owned home, rented/missing status), and the number and ages of other children in the household (coded as three continuous variables: number of children aged 5 years and under, number of children 6–11 years of age, and number of children aged 12–17 other than the respondent). Main income source was categorized as employment (wages, salaries, or self-employment), senior’s income sources (public and private pensions and retirement benefits), dividends and interests, Employment Insurance or workers’ compensation, social assistance, and other/missing.

### Statistical analyses

Descriptive statistics were generated for all sociodemographic variables, estimating means for continuous variables and proportions for categorical variables. Associations between household food insecurity and sociodemographic characteristics were examined using chi-square and Student *t* tests, and bivariate logistic regression analyses were conducted to estimate crude odds ratios. A multivariable logistic regression model was then run including all sociodemographic characteristics to estimate adjusted odds ratios of household food insecurity. Multinomial logistic regression was applied to assess associations between severity of household food insecurity and sociodemographic characteristics, recognizing that moderate and severe food insecurity are much more strongly associated with negative health outcomes than marginal food insecurity (Hutchinson & Tarasuk, 2021; Men et al., 2021a; Ovenell et al., 2022). In all regression analyses, adjusted odds ratios and 95% confidence intervals were estimated with person weights and 1000 bootstrap replicates provided by Statistics Canada. *p*-values less than 0.05 were considered statistically significant.

## Results

The prevalence of household food insecurity was 20.7% among this sample, with 7.3% (CI: 6.4–8.2) of adolescents living in marginally food-insecure households and 13.5% (CI: 12.3–14.5) in moderately or severely food-insecure households. Significant differences were observed in the distributions of all sociodemographic characteristics by household food insecurity status except respondent’s sex and the number of other children 12–17 years of age in the household (Table 1). The mean household income of food-insecure households was about half that of food-secure households ($72,547 versus $136,273).

**Table 1:** Distribution of sociodemographic characteristics among adolescents (*n* = 8416)

	All	Food secure	Food insecure	*p*^*a*^
Sex, %				
Male	51.4	51.5	51.2	0.8831
Female	48.6	48.5	48.8	
Ethno-racial identity, %				
White	63.8	66.9	52.2	<0.0001
Indigenous	5.6	4.6	9.3	
Black	4.5	3.5	8.6	
Other/missing	26.1	25.0	30.0	
Immigration status, %				
Non-immigrant	85.0	86.0	81.4	0.0177
Immigrant, < 5 years	3.5	3.1	5.1	
Immigrant, ≥ 5 years	8.1	7.6	9.9	
Missing	3.4	3.3	3.7	
Province/territory of residence, %				
Newfoundland and Labrador	1.4	1.4	1.2	<0.0001
Prince Edward Island	0.4	0.4	0.4	
Nova Scotia	2.4	2.3	2.6	
New Brunswick	2.0	1.9	2.3	
Quebec	21.0	20.9	21.6	
Ontario	40.1	40.1	40.0	
Manitoba	3.9	4.1	3.1	
Saskatchewan	3.4	3.4	3.3	
Alberta	12.5	12.1	14.4	
British Columbia	12.6	13.2	10.3	
Yukon	0.1	0.1	0.1	
Northwest Territories	0.1	0.1	0.2	
Nunavut	0.2	0.1	0.6	
Rural/urban residence, %				
Urban	81.3	80.6	83.8	0.0062
Rural	18.7	19.4	16.2	
Highest level of education — household, %				
Less than secondary school graduation	3.0	2.3	6.0	<0.0001
Secondary school graduation	10.9	9.1	17.7	
Post-secondary certification	81.4	83.9	71.8	
Missing	4.7	4.8	4.5	
Household income ($CAD), mean ± SEM	123,054 **±** 1973	136,273 ± 2332	72,547 ± 1947	<0.0001
Main source of household income, %				
Employment income	86.7	88.8	78.5	<0.0001
EI/Workers’ compensation	1.0	0.8	2.1	
Social assistance	2.8	1.2	8.8	
Senior benefits	4.3	4.6	3.0	
Other/missing	5.2	4.6	7.6	
Homeownership status, %				
Owned home	76.9	82.6	55.2	<0.0001
Rented/missing status	23.1	17.4	44.8	
Living arrangement, %				
Living with two parents	70.2	74.7	53.0	<0.0001
Living with single parent	19.2	15.4	33.8	
Other living arrangement	10.6	9.9	13.3	
Number of children ≤5 y, mean ± SEM	0.12 **±** 0.01	0.09 **±** 0.01	0.21 **±** 0.02	<0.0001
Number of children 6–11 y, mean ± SEM	0.43 **±** 0.01	0.41 **±** 0.01	0.49 **±** 0.03	0.0086
Number of children 12–17 y other than respondent, mean ± SEM	0.55 **±** 0.01	0.54 ± 0.01	0.60 ± 0.03	0.0658

^*a*^*p*-value based on chi-square test for categorical variables and the Student *t* test for differences in means between the food secure and food insecure for continuous variables

The prevalence of household food insecurity differed markedly among adolescents depending on their ethno-racial identity, socioeconomic circumstances, and province or territory of residence (Table 2). Food insecurity was most prevalent among adolescents identifying as Black (39.2%) or Indigenous (34.3%), those living in households reliant on social assistance (65.3%) or Employment Insurance or workers’ compensation (42.6%), those living in a rental accommodation (40.3%), those living with a single parent (36.5%), and those in households where the highest level of education was secondary school (33.6%) or lower (41.0%) (Table 2). Across the country, food insecurity prevalence ranged from 16.3% in Manitoba to 72.0% in Nunavut (Table 2). There was no difference in prevalence in relation to the respondent’s sex and relatively small differences in relation to their immigration status and rural versus urban residence.

**Table 2:** Household food insecurity status by sociodemographic characteristic

Characteristic	Food secure (%)	Food insecure (%)			*p*^*a*^
		Marginal	Moderate-severe	Any food insecurity	
Sex					
Male	79.4	6.9	13.8	20.6	0.5348
Female	79.2	7.8	13.1	20.9	
Ethno-racial identity					
White	83.0	6.3	10.7	17.0	<0.0001
Indigenous	65.7	7.2	27.2	34.3	
Black	60.8	8.8	30.4	39.2	
Other/missing	76.1	9.6	14.3	23.9	
Immigration status					
Non-immigrant	80.1	6.8	13.1	19.9	0.0197
Immigrant, < 5 years	70.1	14.3	15.6	29.9	
Immigrant, ≥ 5 years	74.7	10.2	15.1	25.3	
Missing	77.6	7.4	15.0	22.4	
Province/territory of residence					
Newfoundland and Labrador	81.5	4.3	14.2	18.5	<0.0001
Prince Edward Island	80.4	5.1	14.5	19.6	
Nova Scotia	77.7	5.4	17	22.3	
New Brunswick	75.7	4.4	19.9	24.3	
Quebec	78.7	8.8	12.5	21.3	
Ontario	79.3	7.5	13.2	20.7	
Manitoba	83.8	6.6	9.6	16.3	
Saskatchewan	79.5	6.3	14.1	20.5	
Alberta	76.2	7.6	16.2	23.8	
British Columbia	83.0	5.5	11.4	17.0	
Yukon	81.7	5.1	13.2	18.3	
Northwest Territories	65.8	7.0	27.2	34.2	
Nunavut	28.0	8.0	64.0	72.0	
Urban/rural residence					
Population centre	78.6	7.6	13.8	21.4	0.0286
Rural	82.1	6.3	11.6	17.9	
Highest level of education — household					
Less than secondary school graduation	59.0	8.5	32.5	41.0	<0.0001
Secondary school graduation	66.4	11.7	22.0	33.6	
Post-secondary certification	81.7	6.7	11.6	18.3	
Missing	80.1	6.8	13.1	19.9	
Main source of household income					
Employment income	81.2	7.1	11.7	18.8	<0.0001
EI/Workers’ compensation	57.4	7.1	35.6	42.6	
Social assistance	34.7	12.7	52.7	65.3	
Senior benefits	85.4	6.6	8.0	14.6	
Other/missing	69.9	8.5	21.6	30.1	
Homeownership status					
Owned home	85.1	6.2	8.7	14.9	<0.0001
Rented/missing status	59.7	11.2	29.1	40.3	
Living arrangement					
Living with two parents	84.3	6.0	9.7	15.7	<0.0001
Living with single parent	63.5	11.0	25.5	36.5	
Other living arrangement	74.1	9.4	16.5	25.9	

Note: Row percentages may not add to 100% due to rounding

^*a*^Chi-square *p*-value indicates differences in distribution of household food insecurity (any food insecure vs food secure) for each sociodemographic characteristic

Table 3 shows the crude and adjusted odds ratios of household food insecurity for the sociodemographic characteristics considered. The adjusted odds of food insecurity were significantly elevated among adolescents living with a single parent or without their parents (other/missing), those living with a greater number of children ≤ 5 years, those in rented accommodation, those in households where the highest level of education was secondary school, those in households reliant on social assistance, and those identifying as Black or Indigenous. The crude odds associated with an increasing number of 12- to 17-year-olds other than the respondent was not statistically significant, but it became significant after adjustment for other characteristics (aOR: 1.25, CI: 1.11–1.40). Higher income was protective against household food insecurity; the adjusted odds of household food insecurity fell by 1% (CI: 0.99–0.99) with each additional $1000.

**Table 3:** Crude and adjusted odds ratios (95% CI) of household food insecurity among adolescents for sociodemographic characteristics

Characteristic	Crude OR (95% CI)	Adjusted OR (95% CI) ^*a*^
Sex		
Female	1.01 (0.86–1.20)	1.05 (0.87–1.25)
Male (ref)	1.00	1.00
Ethno-racial identity		
White (ref)	1.00	1.00
Indigenous	2.56 (2.01–3.26) **	1.80 (1.37–2.35) **
Black	3.16 (2.13–4.68) **	1.80 (1.11–2.94) *
Other/missing	1.54 (1.26–1.87) **	1.29 (0.99–1.68)
Province/territory of residence		
British Columbia	0.78 (0.60–1.02)	0.78 (0.58–1.04)
Alberta	1.20 (0.94–1.52)	1.48 (1.13–1.94) *
Saskatchewan	0.98 (0.70–1.39)	1.00 (0.69–1.44)
Manitoba	0.74 (0.52–1.06)	0.66 (0.44–1.00) *
Ontario (ref)	1.00	1.00
Quebec	1.04 (0.83–1.29)	0.92 (0.72–1.19)
Newfoundland	0.87 (0.59–1.28)	1.16 (0.76–1.79)
New Brunswick	1.23 (0.85–1.77)	1.34 (0.91–1.97)
Nova Scotia	1.10 (0.78–1.55)	1.17 (0.80–1.72)
Prince Edward Island	0.94 (0.61–1.43)	1.08 (0.69–1.70)
Yukon	0.86 (0.53–1.39)	0.96 (0.53–1.74)
Northwest Territories	1.99 (1.17–3.38) *	2.11 (1.09–4.09) *
Nunavut	9.83 (4.87–19.85) **	6.77 (2.75–16.66) **
Rural vs. urban area of residence		
Urban area (ref)	1.00	1.00
Rural area	0.80 (0.68–0.94) **	0.89 (0.74–1.07)
Highest level of education		
Less than secondary school graduation	3.10 (2.09–4.59) **	0.96 (0.59–1.56)
Secondary school graduation	2.26 (1.78–2.87) **	1.38 (1.05–1.82) *
Post-secondary certification (ref)	1.00	1.00
Missing	1.11 (0.72–1.72)	0.78 (0.47–1.29)
Immigration status		
Immigrant (<5 years)	1.72 (1.08–2.74) *	0.92 (0.53–1.60)
Immigrant (≥5 years)	1.37 (1.00–1.87)	0.96 (0.64–1.45)
Non-immigrant (ref)	1.00	1.00
Other/missing	1.17 (0.76–1.80)	0.60 (0.35–1.02)
Homeownership status		
Owned (ref)	1.00	1.00
Rented/missing	3.86 (3.23–4.60) **	1.98 (1.59–2.46) **
Household income, before tax	0.99 (0.99–0.99) **	0.99 (0.99–0.99) **
Main source of household income		
Employment income (ref)	1.00	1.00
Senior’s benefits (including dividends/interest)	0.74 (0.47–1.17)	0.74 (0.46–1.17)
EI/Workers’ compensation	3.21 (1.73–5.96) **	1.30 (0.66–2.56)
Social assistance	8.14 (5.31–12.50) **	2.03 (1.27–3.24) *
Other/missing	1.86 (1.28–2.73) *	0.82 (0.56–1.18)
Respondent living arrangement		
Living with two parents (ref)	1.00	1.00
Living with single parent	3.09 (2.55–3.74) **	1.60 (1.28–1.98) **
Other/missing	1.88 (1.45–2.44) **	1.49 (1.09–2.05) *
Number of children aged ≤ 5 (ref: no children aged ≤ 5)	1.84 (1.52–2.21) **	1.45 (1.18–1.78) **
Number of children aged 6–11 (ref: no children aged 6–11)	1.19 (1.05–1.34) *	1.07 (0.94–1.22)
Number of children aged 12–17 (other than respondent; ref: no other children 12–17)	1.13 (1.00–1.27)	1.25 (1.11–1.40) **

^*a*^Adjusted for all other variables in the model

*p < 0.05, **p < 0.001

The crude odds of living in a food-insecure household only differed from Ontario for residents of the Northwest Territories and Nunavut, but differences were also observed for Manitoba and Alberta when other sociodemographic characteristics were taken into account (Table 3). Compared to adolescents in Ontario, adolescents in Nunavut had almost seven times the odds (aOR: 6.77, CI: 2.75–16.66) of living in food-insecure households and those in Northwest Territories had double the odds (aOR: 2.11, CI: 1.09–4.09) after adjusting for other sociodemographic characteristics. Adolescents in Alberta also had a significantly higher adjusted odds of household food insecurity (aOR: 1.48, CI: 1.13–1.94), whereas adolescents in Manitoba had lower adjusted odds of food insecurity (aOR: 0.66, CI: 0.44–1.00) compared to adolescents in Ontario.

When we repeated our multivariable analyses using multinomial logistic regression to differentiate marginal from moderate or severe household food insecurity, only income, housing tenure, education, and living arrangements were significantly associated with marginal food insecurity (Supplementary material, Appendix 1). A much broader array of characteristics was associated with moderate/severe household food insecurity, with findings generally similar to those observed for food insecurity in Table 3 and most adjusted odds ratios slightly higher in magnitude (Supplementary material, Appendix 1). In the multi-nomial model, we found no significant difference in the adjusted odds of moderate/severe food insecurity for adolescents in Manitoba versus Ontario (aOR: 0.60, CI: 0.35–1.04), but significantly elevated odds for adolescents in New Brunswick (aOR: 1.77, CI: 1.15–2.74) as well as Alberta (aOR: 1.67, CI: 1.22–2.29), Northwest Territories (aOR: 2.52, CI: 1.25–5.07), and Nunavut (aOR: 8.42, CI: 3.11–22.79).

## Discussion

Our findings indicate that household food insecurity among adolescents is tightly linked to their ethno-racial identity, province or territory of residence, household structure, and household economic status. We found that Black or Indigenous identity, lower income, reliance on social assistance, lower household education, living in a dwelling that was rented rather than owned, not living with two parents, and living in a household with a greater number of young children or adolescents were significant predictors of household food insecurity among adolescents. Our results extend the understanding of food insecurity that has emerged from studies of the general population (McIntyre et al., 2014; McIntyre et al., 2016b; Tarasuk et al., 2019a) by highlighting differences in risk specific to adolescents and the households in which they live.

Our results are correlational, not causal, but the observed inverse association between household income and household food insecurity is consistent with Canadian research showing the positive effects of policy interventions that improve households’ finances on food insecurity prevalence and severity (Brown & Tarasuk, 2019; Ionescu-Ittu et al., 2015; Loopstra et al., 2015; McIntyre et al., 2016a; Men et al., 2021c). However, reducing the prevalence of food insecurity has so far not been an explicit policy objective at any level of government in Canada, so the policy effects observed in these studies have been incidental to other goals, not the result of deliberate interventions.

The public policy of most relevance to our findings is the Canada Child Benefit (CCB) because this federal income transfer, introduced in 2016, reaches almost all families with children under 18. A higher benefit is provided for children under 6 than those 6–17 years of age, and the total amount is reduced as family net income rises. Although the CCB has been indexed to inflation (Department of Finance Canada, 2018), benefit amounts are uniform across the country with no allowances for regional differences in costs of living. The CCB has been widely credited with reducing child poverty, but its only observed effect on household food insecurity was a modest reduction in the prevalence of severe food insecurity following its initial implementation (Brown & Tarasuk, 2019). It is noteworthy, however, that the greatest reduction in severe food insecurity was observed among low-income families (Brown & Tarasuk, 2019). This is consistent with findings from a large international comparative study showing that more generous cash transfer programs for families with children have the greatest impact on food insecurity among families at the bottom of the income distribution and reduce the most severe forms of food insecurity (Reeves et al., 2021). Yet our results indicate that food insecurity remains a serious problem for Canadian families.

More research is required to understand how the CCB could be structured to better protect families with children from food insecurity, but the strong relationship between household incomes and food insecurity documented in this study of adolescents suggests a need to increase the benefit for low-income families with children of all ages. The extraordinary vulnerability of families in Nunavut raises questions about the federal government’s logic for not taking major regional differences in costs of living into account when setting benefit amounts. Additionally, the very high rates of food insecurity among single-parent families, families reliant on social assistance, and those who rent their accommodation suggest that these population subgroups could benefit from more support. Our finding of higher adjusted odds of household food insecurity with an increasing number of children aged ≤ 5 or 12–17 years also point to potentially important differences in the needs of families with children in different life stages that impact food insecurity risk. If confirmed through further investigation, this could also inform the design of the CCB relative to children’s ages.

That most households dependent on social assistance programs in Canada are food insecure is a well-established fact (McIntyre et al., 2016b; Tarasuk & Mitchell, 2020; Tarasuk et al., 2019a; Dhunna & Tarasuk, 2021; Polsky & Garriguet, 2022). Our findings add to this literature by revealing the pervasive exposure to household food insecurity among 12–17-year-olds in families reliant on social assistance. The data available for social assistance in CCHS do not permit differentiation of general welfare from disability support programs, so we are unable to determine the food insecurity status of families in relation to these different programs. The four-fold drop in the odds associated with social assistance following adjustment for income, housing tenure, living arrangements, and other sociodemographic characteristics highlights the strong intersection of these income support programs with other dimensions of financial precarity. Administered by the provinces and territories, social assistance programs are typically characterized by low benefit levels and stringent income and asset eligibility criteria that ensure recipients are divested of any assets and savings. The food insecurity status of social assistance recipients has been shown to be very sensitive to increases in benefits and other program changes that improve recipients’ financial circumstances (Loopstra et al., 2015), but benefits remain well below poverty levels and in most jurisdictions they are not indexed to inflation.

In contrast to prior Canadian research (Tarasuk et al., 2019a), we did not observe a significantly lower odds of household food insecurity among adolescents living in households reliant on senior’s incomes and/or dividends and interest. While the income adequacy and security afforded to Canadian seniors though public pensions are believed to account for the very low rates of household food insecurity observed among this age group (McIntyre et al., 2016a), the reliance on senior’s income sources by households including adolescents connotes no similar advantage, perhaps because public old-age pensions are not designed to support extended families.

Renting rather than owning one’s home has long been identified as an independent predictor of household food insecurity in Canada, with more in-depth examinations suggesting that this finding relates to the greater wealth associated with home ownership (McIntyre et al., 2016b; Fafard St-Germain & Tarasuk, 2020). The main approach to improve housing affordability among low-income renters in Canada has been subsidized housing, but access to such housing is limited (Government of Canada, 2017), and the very high rates of food insecurity observed among households in government-subsidized housing (Fafard St-Germain & Tarasuk, 2017) suggest that these programs provide insufficient support to enable many residents to achieve household food security. The federal government has committed to improving housing affordability (Government of Canada, 2017), but as this national housing strategy unfolds, it will be important to monitor its effects on household food insecurity.

Even after adjusting for important predictors of food insecurity such as low income, renting, and lower educational attainment, adolescents who identified as Black or Indigenous had 80% higher odds of living in a food-insecure household compared to those who identified as white. This too has been documented in prior Canadian research (Dhunna & Tarasuk, 2021; Tarasuk et al., 2019b; McIntyre et al., 2014), and it points to the strong influences of structural racism and colonialism in shaping households’ food security. Racialized individuals have higher rates of unemployment and when in the workforce, have greater likelihood of precarious employment, low wages, and a lack of benefits (Block & Galabuzi, 2018; Lewchuk et al., 2015; Moyser, 2017). Initiatives that target structural racism in Canada’s labour market may be critical to addressing household food insecurity among racialized adolescents. Additionally, our results lend support to calls for the Government of Canada to redress the legacies of colonialism and help facilitate Indigenous food sovereignty.

The extremely high prevalence and markedly elevated odds of household food insecurity among adolescents in Nunavut compared to adolescents in Ontario indicate even greater disparities than we observed in an earlier analysis of Canadian households (Tarasuk et al., 2019a). How much this reflects worsening conditions in Nunavut versus the heightened vulnerability of adolescents living there is unclear, but our results lend support to the urgent calls for more effective responses to food insecurity among northern and Indigenous populations (Inuit Tapiriit Kanatami, 2021).

The other inter-jurisdictional differences observed in this study were of much smaller magnitude than those for Nunavut, but the significantly higher adjusted odds of moderate/severe food insecurity among adolescents living in Alberta, New Brunswick, and Northwest Territories compared to Ontario also suggest potentially important differences in provincial and territorial policies that merit more investigation. Evidence that food insecurity rates among families with children are lower in provinces with higher minimum wages and welfare benefits and lower income tax rates for the lowest income households (Men et al., 2021c) highlights the sensitivity of household food insecurity to provincial policy actions.

### Strengths and limitations

Strengths of this study include the use of a large, population-representative sample, a well-validated measure of food insecurity, and simultaneous adjustment for multiple sociodemographic characteristics. However, we were unable to account for some variables associated with increased risk of food insecurity, including parental employment and health or disability status. Our study is also limited by the fact that we used data from 2017 to 2018. Although household food insecurity has been measured on more recent cycles of CCHS and the Canadian Income Survey (CIS), we elected to use CCHS 2017–2018 because of pandemic-related disruptions to subsequent data collections and the lack of population-representative data for the territories on more recent surveys (Polsky & Garriguet, 2022). A further limitation of CCHS and CIS is that neither survey includes individuals living on First Nations reserves. Thus, our results with respect to Indigenous identity only apply to adolescents living off reserves. It is also important to emphasize that our analysis is correlational, based on cross-sectional data covering a 12-month period. Longitudinal studies are needed to identify the family characteristics and circumstances that precipitate or mitigate household food insecurity. Longitudinal data are also badly needed to determine the duration of adolescents’ exposures to household food insecurity, recognizing that the implications for their health and well-being must be a function of both the severity and chronicity of the exposure.

## Conclusion

Given the serious negative health implications of household food insecurity for adolescents, the high prevalence and heightened vulnerability observed among select subpopulations highlight the need for policies that will better protect families with adolescents from household food insecurity.

## Contributions to knowledge

What does this study add to existing knowledge?

- Household food insecurity has serious negative health implications for adolescents, and it affected one in five adolescents in Canada in 2017–2018.
- We found that Black or Indigenous identity, lower income, reliance on social assistance, lower household education, living in rented accommodation, not living with two parents, and living in a household with a greater number of young children or adolescents were all significant predictors of household food insecurity among adolescents.
- Risk was highest for adolescents in Nunavut, but also elevated for adolescents in Alberta, New Brunswick, and Northwest Territories compared to Ontario.

What are the key implications for public health interventions, practice, or policy?

- Our results lend support to calls for more deliberate, evidence-based policy interventions by the federal, provincial, and territorial governments to reduce food insecurity among families with children.
- Consideration should be given to improving the Canada Child Benefit for low-income families and providing enhanced benefits to families in Nunavut, single-parent families, and those who rent their accommodation.
- Measures to address structural racism and colonialism are critical to supporting food security for Black and Indigenous adolescents.
- Reforms to provincial/territorial social assistance programs are urgently needed to enable recipients to meet their basic needs for food.

## Supplementary Information

ESM 1 (DOCX 39 kb)

## Data Availability

The data analyzed in this study can be accessed through the Statistics Canada Research Data Centres for researchers who meet the criteria for access to confidential data.

## Code availability

All programming was performed with SAS. SAS code is available from the authors upon request.
