# Supplementary material for: The prevalence and predictors of household food insecurity among adolescents in Canada
Source: Can J Public Health. 2023 Jan 23;114(3):453–63. doi: 10.17269/s41997-022-00737-2 (PMC10156911; doi:10.17269/s41997-022-00737-2)
Supplement: Supplementary file 1 — (DOCX 39 kb) [file 41997_2022_737_MOESM1_ESM.docx]

**Appendix 1:**

Adjusted odds (95% CI) of marginal and moderate-severe household food insecurity in relation to sociodemographic characteristics among adolescents

| **Characteristic** | **Adjusted OR (95% CI) *^a^*** | |
| --- | --- | --- |
|  | **Marginal food insecurity *^b^*** | **Moderate - severe food insecurity *^b^*** |
| **Sex** |  |  |
| Female | 1.16 (0.89 - 1.52) | 0.99 (0.80 - 1.22) |
| Male (ref) | 1.00 | 1.00 |
|  |  |  |
| **Ethnoracial identity** |  |  |
| White (ref) | 1.00 | 1.00 |
| Indigenous | 1.22 (0.83 - 1.80) | 2.11 (1.52 - 2.93) ** |
| Black | 1.22 (0.53 - 2.81) | 2.16 (1.27 - 3.68) * |
| Other/missing | 1.39 (0.94 - 2.06) | 1.22 (0.89 - 1.67) |
|  |  |  |
| **Province/territory of residence** |  |  |
| British Columbia | 0.66 (0.43 - 1.01) | 0.86 (0.61 - 1.23) |
| Alberta | 1.23 (0.80 - 1.89) | 1.67 (1.22 - 2.29) * |
| Saskatchewan | 0.92 (0.49 - 1.73) | 1.05 (0.67 - 1.64) |
| Manitoba | 0.76 (0.44 - 1.29) | 0.60 (0.35 - 1.04) |
| Ontario (ref) | 1.00 | 1.00 |
| Quebec | 1.13 (0.78 - 1.63) | 0.81 (0.60 - 1.10) |
| Newfoundland | 0.75 (0.35 - 1.62) | 1.45 (0.89 - 2.37) |
| New Brunswick | 0.67 (0.33 - 1.38) | 1.77 (1.15 - 2.74) * |
| Nova Scotia | 0.80 (0.41 - 1.59) | 1.41 (0.89 - 2.23) |
| Prince Edward Island | 0.73 (0.27 - 1.99) | 1.34 (0.76 - 2.34) |
| Yukon | 0.78 (0.25 - 2.42) | 1.08 (0.52 - 2.27) |
| Northwest Territories | 1.50 (0.31 - 7.33) | 2.52 (1.25 - 5.07) * |
| Nunavut | 3.56 (0.75 - 16.90) | 8.42 (3.11 - 22.79) ** |
|  |  |  |
| **Urban/rural residence** |  |  |
| Population centre (ref) | 1.00 | 1.00 |
| Rural area | 0.94 (0.70 - 1.25) | 0.86 (0.69 - 1.08) |
|  |  |  |
| **Highest level of education** |  |  |
| Less than secondary school graduation | 0.76 (0.33 - 1.78) | 1.04 (0.59 - 1.80) |
| Secondary school graduation | 1.56 (1.01 - 2.40) * | 1.30 (0.97 - 1.75) |
| Post-secondary certification (ref) | 1.00 | 1.00 |
| Missing | 0.73 (0.30 - 1.79) | 0.81 (0.46 - 1.45) |
|  |  |  |
| **Immigration status** |  |  |
| Immigrant (<5 years) | 1.48 (0.67 - 3.27) | 0.67 (0.36 - 1.25) |
| Immigrant (≥5 years) | 1.22 (0.71 - 2.08) | 0.83 (0.50 - 1.37) |
| Non-immigrant (ref) | 1.00 | 1.00 |
| Other/missing | 0.67 (0.28 - 1.60) | 0.57 (0.31 - 1.05) |
|  |  |  |
| **Homeownership status** |  |  |
| Owned (ref) | 1.00 | 1.00 |
| Rented/missing | 1.49 (1.04 - 2.15) * | 2.29 (1.79 - 2.92) ** |
|  |  |  |
| **Household income, before tax** | 0.99 (0.99 - 1.00) ** | 0.99 (0.99 - 0.99) ** |
|  |  |  |
| **Main source of household income** |  |  |
| Employment income (ref) | 1.00 | 1.00 |
| Senior's benefits (incl. div/interests) | 0.90 (0.39 - 2.10) | 0.64 (0.38 - 1.07) |
| EI/Workers’ Comp. | 0.66 (0.10 - 4.36) | 1.60 (0.75 - 3.40) |
| Social assistance | 1.47 (0.73 - 2.97) | 2.21 (1.34 - 3.67) * |
| Other/missing | 0.77 (0.42 - 1.42) | 0.82 (0.52 - 1.28) |
|  |  |  |
| **Respondent living arrangement** |  |  |
| Living with two parents (ref) | 1.00 | 1.00 |
| Living with single parent | 1.54 (1.10 - 2.16) * | 1.61 (1.25 - 2.08) ** |
| Other/missing | 1.61 (1.01 - 2.55) * | 1.44 (1.01 - 2.05) * |
|  |  |  |
| **Number of children aged ≤5** | 1.19 (0.91 - 1.56) | 1.57 (1.23 - 2.02) ** |
|  |  |  |
| **Number of children aged 6-11** | 0.98 (0.79 - 1.21) | 1.13 (0.97 - 1.31) |
|  |  |  |
| **Number of children aged 12-17** (other than respondent) | 1.19 (0.99 - 1.44) | 1.28 (1.11 - 1.46) ** |

*^a^* Adjusted for all other variables in the model

*^b^* Reference group: food secure.

* p <0.05, ** p < 0.001
